# Evaluation of Serum Adipocytokines and Cystatin C As Early Biomarkers in Chronic Kidney Disease Patients and Their Relation to Endothelial Dysfunction

**DOI:** 10.7759/cureus.109069

**Published:** 2026-05-18

**Authors:** Harish G Bagewadi, Sachin Patil, Manjushree Sugoor, Renuka S Melkundi, Shivalee A, Rohini Kallur, Sangamesh B Tondare

**Affiliations:** 1 Pharmacology, Gulbarga Institute of Medical Sciences, Kalaburagi, IND; 2 General Medicine, Gulbarga Institute of Medical Sciences, Kalaburagi, IND; 3 Biochemistry, Gulbarga Institute of Medical Sciences, Kalaburagi, IND; 4 Otolaryngology, Gulbarga Institute of Medical Sciences, Kalaburagi, IND; 5 Research, Gulbarga Institute of Medical Sciences, Kalaburagi, IND; 6 General Surgery, Gulbarga Institute of Medical Sciences, Kalaburagi, IND

**Keywords:** chronic kidney disease (ckd), cystatin c, endothelial dysfunction, endothelial nitric oxide synthase (enos), leptin adiponectin, nitric oxide synthase, serum adipocytokines

## Abstract

Background

Chronic kidney disease (CKD) is strongly associated with an increased incidence of cardiovascular complications. One of the mechanisms contributing to vascular damage in CKD is the decreased availability of nitric oxide (NO), an important regulator of vascular tone and endothelial function. The adipocytokines have been suggested to play a significant role in the vascular abnormalities observed in patients with impaired renal function.

Methods

This study included 81 patients diagnosed with CKD who were classified into three groups based on their estimated glomerular filtration rate (eGFR). Group I (n=27) consisted of patients with eGFR values ranging from 60-90 ml/min/1.73 m² (CKD stages I-II). Whereas, group II (n=27) included patients with eGFR levels between 15-59 ml/min/1.73 m² (stages III-IV), while group III (n=27) comprised patients with eGFR values below 15 ml/min/1.73 m² (stage V). The serum concentrations of adiponectin, leptin, cystatin C, and endothelial nitric oxide synthase (eNOS) were measured using commercially available enzyme-linked immunosorbent assay (ELISA) kits (Elabscience, Houston, Texas, USA).

Results

In the present study, the serum levels of adiponectin, leptin, and cystatin C increased progressively from group I to group III, which was statistically significant (p<0.05). In contrast, serum eNOS levels showed a decreasing trend with advancing stages of kidney disease. The random blood sugar (RBS) and serum creatinine levels were significantly higher (p<0.05) in patients belonging to group III compared with group II and group I. The correlation analysis revealed a strong negative association between eGFR and serum eNOS levels in groups III, II, and I (r= −0.72, −0.56, and −0.22, respectively).

Conclusion

The findings of this study indicate that worsening renal function in CKD is associated with significant alterations in circulating adiponectin, leptin, and cystatin C levels. These biochemical changes, together with reduced eNOS activity, may contribute to endothelial dysfunction and could increase the risk of cardiovascular complications in patients with CKD.

## Introduction

Chronic kidney disease (CKD) has become an important global health concern, affecting nearly 11-13% of the adult population worldwide [1-2]. In individuals with impaired renal function, disturbances in adipokine secretion as well as increased production of inflammatory cytokines may enhance vascular inflammation and endothelial injury, thereby contributing to the elevated cardiovascular risk associated with CKD [3]. Among the various adipokines involved in metabolic and inflammatory regulation, adiponectin has received considerable attention for its potential role in renal disease. Adiponectin is known to exert anti-inflammatory, antioxidant, and insulin-sensitizing effects through its receptors, Adiponectin Receptor 1 and 2 (ADIPOR1, ADIPOR2), and T-cadherin, which are widely expressed in tissues such as the liver, kidneys and skeletal muscles [4]. Leptin is an adipocyte-derived hormone that has been linked to derangement of renal function. As kidneys play a vital role in leptin clearance, the concentrations of leptin tend to increase progressively as kidney impairment advances [5]. Cystatin C is increasingly recognized as a sensitive indicator of kidney function. In contrast to serum creatinine, its levels are less influenced by variables such as muscle mass, diet, and sex, which enhance its reliability in specific clinical contexts [6]. Although interest in adipokines in CKD has grown, the precise relationship between these biomarkers and the progression of renal impairment is not yet fully clarified.

This study aimed to: 1) estimate the serum levels of adiponectin and leptin; 2) assess the serum levels of cystatin C and endothelial nitric oxide (eNOS); and 3) assess the correlation of serum levels of adiponectin, leptin, and cystatin C with eNOS levels in patients with CKD.

## Materials and methods

Patient recruitment

This was a hospital-based, single-center, cross-sectional study. The study included participants recruited from the outpatient and inpatient units of the Department of General Medicine and was conducted from January 2025 to February 2026 [7]. The approval was granted by the Institutional Ethics Committee prior to the study (approval no: GIMS/KLB/PHARMA/IEC/291/2023-24; dated 08-07-2024). All procedures were adhered to institutional and ethical guidelines, and written informed consent was secured from each participant before enrolment.

Inclusion Criteria

Individuals of both sexes, aged between 30 and 70 years, who had been clinically diagnosed with CKD ranging from stage I to stage V were included in the study. Hypertensive patients and those with diabetes were also included in the study. 

Exclusion Criteria

Patients receiving haemodialysis, peritoneal dialysis, those with a history of organ transplantation, or individuals on long-term immunosuppressive therapy were excluded from participation. Patients with alcoholic heart disease were not involved in the study. 

A total of 81 patients diagnosed with CKD were recruited for the present study. The estimated glomerular filtration rate (eGFR) was estimated using the Cockcroft-Gault equation based on serum creatinine levels [8]. They were then categorized into three groups: Group I (n=27): patients with mild renal impairment corresponding to CKD stages I-II, with eGFR values ranging from 60 to 90 ml/min/1.73 m² [9]; Group II (n=27): patients with moderate-to-severe renal dysfunction, classified as CKD stages III-IV, with eGFR values between 15 and 59 mL/min/1.73 m²; and Group III (n=27): patients with end-stage renal disease representing CKD stage V, with eGFR values below 15 ml/min/1.73 m². Using Epi Info software (version 7.1, Centers for Disease Control and Prevention), the sample size was determined according to the method described by Younes et al. [2,10]. A prevalence of 10% for CKD was adopted based on the report by Mohanty et al. [11]. The calculation was based on a 95% confidence level, 80% statistical power, and a 6% margin of error (d=0.06), with an alpha (α) value of 0.05. Using the standard formula: N = Z2 x p x (1-p) / d2 [12], where p=0.10, (1-p) =0.90, and Z=1.96, the final computed sample size was 81 participants. In addition, demographic and background information, including age, sex, BMI, and smoking habits, were documented for all study participants.

Data collection

Informed consent was obtained prior to sample collection. Clinical data and blood specimens were obtained at the multidisciplinary research unit, where approximately 5 mL of venous blood was collected from each participant using sterile procedures. The collected samples were centrifuged at 3000 rpm for 10 minutes, after which the serum was separated and stored at 2-8 °C until further biochemical analysis. The parameters assessed included random blood sugar (RBS), serum creatinine, and components of the lipid profile, namely high-density lipoprotein (HDL), low-density lipoprotein (LDL), and triglycerides [7].

Serum concentrations of specific biomarkers were determined using commercially available human enzyme-linked immunosorbent assay (ELISA) kits procured from the manufacturer (Elabscience, Houston, Texas, USA). The assays were performed according to the manufacturer’s instructions using the following kits: adiponectin (Cat. No. E-EL-H6122, sensitivity: 0.1ng/mL, detection range: 0.16-10 ng/mL); leptin (Cat. No. E-EL-H6017, sensitivity: 9.38pg/mL, detection range: 15.63-1000 pg/mL); cystatin C (Cat. No. E-EL-H3643, sensitivity: 0.19 ng/mL, detection range: 0.31-20 ng/mL) [2]; and eNOS (Cat. No. E-EL-H0755; sensitivity: 37.5 pg/mL; detection range: 62.5-4000 pg/mL). The intra- and inter-assay coefficients of variation were maintained below 10%.

Statistical analysis

The analysis was performed using IBM SPSS Statistics for Windows, Version 16 (Released 2007; IBM Corp., Armonk, New York, United States). The continuous variables were expressed as mean ± standard deviation (SD), whereas categorical variables were presented as frequencies and percentages. The chi-square test was applied to compare categorical variables between the study groups [13]. The differences in serum biomarker levels (leptin, adiponectin, cystatin C, and eNOS among CKD groups were assessed using one-way analysis of variance (ANOVA) for continuous variables. When overall significance was detected, Tukey’s post hoc test was applied to identify pair wise group differences. Associations between variables were examined using Pearson’s correlation coefficient. A p-value <0.05 was considered statistically significant.

## Results

Table 1 presents the demographic and clinical characteristics of the study participants.

**Table 1:** Demographic characteristics of the study population *p <0.05 versus group I, ^†^p <0.01 versus group I, ^‡^p <0.05 versus group II.  Data are presented as numbers, Mean ± Standard Deviation; chi-square test (χ² -chi square value), and ANOVA (F-test statistic) was applied. The p-value with* indicates statistical significance (<0.05). BMI: Body mass index; SBP: systolic blood pressure; DBP: diastolic blood pressure.

Variables	Group I	Group II	Group III	Test value	p-value
Age (Years)	48.32±1.38	52.20±0.34	50.16±0.42	F = 2.766	0.723
Male: Female	20:7	16:11	18:09	χ² = 2.059	0.325
Smoking: non-smoking	15:12	19:08*	20:07*	χ² = 2.142	0.038
BMI -kg/m²	36.4±1.72	41.8±4.36*	43.8±4.36^†^	F = 11.141	0.032
SBP (mm/Hg)	136.02±2.3	150.76±1.8*	180.76±1.4^†,^^‡^	F = 13.348	0.041
DBP (mm/Hg)	82.64±1.6	92.84±2.4*	110.84±2.8^†,^^‡^	F = 12.176	0.026

The mean age of patients in group I, group II, and group III was 48.32 ± 1.38 years, 52.20 ± 0.34 years, and 50.16 ± 0.42 years, respectively. A predominance of male participants was observed across all groups compared with female subjects. The prevalence of smoking was higher among patients in group III and group II compared with those in group I. In addition, a significant increase in systolic blood pressure, diastolic blood pressure, and BMI was observed in group III and group II patients when compared with group I.

Among CKD patients, 9.9% (n=8) were classified as Stage I, 23.5% (n=19) as Stage II, 22.2% (n=18) as Stage III and 11.1% (n=9) as Stage IV and 33.3% (n=27) as Stage V (Table 2) [14].

**Table 2:** Showing the counts, percentages, and stages of patients with CKD Data are presented as numbers and percentages. eGFR: estimated Glomerular filtration rate.

Stages of CKD	Descriptive - eGFR (ml/min/1.73 m^2^)	N (%)
Stage I	>90	8 (9.9%)
Stage II	60-89	19 (23.5%)
Stage III	30-59	18 (22.2%)
Stage IV	15-29	9 (11.1%)
Stage V	<15	27 (33.3%)

Table 3 shows the comparison of biochemical parameters among the study groups.

**Table 3:** The comparison of serum creatinine, TG, LDL, HDL levels in patients with CKD Data are presented as mean ± standard deviation; ANOVA was applied. Test statistic (f) -value, *p<0.05 versus group I, ^†^p<0.01 versus group I, ^‡^p<0.05 versus group II. S: serum; TG: triglycerides; LDL: low-density lipoprotein; HDL: high-density lipoprotein; CKD: chronic kidney disease.

Parameters	Group I	Group II	Group III	(F)-value	p- value
S. creatinine (mg/dL)	1.7 ±1.53	2.5 ±1.70*	3.8±1.58^†,‡^	96.23	0.041
RBS (mg/dL)	280.9 ±1.55	330.2 ±1.90*	380.8±1.72^†,‡^	56.52	0.018
S. TG (mg/dL)	242±3.5	285±2.1*	296±8.4^†,‡^	24.24	0.036
S. LDL (mg/dL)	145±4.31	155±2.45*	185±3.38^†,‡^	32.86	0.042
S. HDL (mg/dL)	32.4±1.44	30.2±1.38*	24.5±1.25^†,‡^	28.48	0.024

The serum creatinine levels were significantly elevated in group III and group II patients compared with those in group I. Similarly, random blood sugar (RBS) levels were significantly higher in group III and group II than in group I. The lipid profile analysis revealed significantly increased levels of serum triglycerides and LDL in patients belonging to group III and group II compared with group I. In contrast, HDL levels were lower in group III and group II patients than in those in group I.

Table 4 illustrates the comparison of serum biomarker levels among the study groups.

**Table 4:** The comparison of serum adiponectin, leptin, cystatin C, and eNOS levels in patients with CKD Data are presented as mean ± standard deviation; ANOVA was applied. *p<0.05, versus group I, ^†^p<0.01 versus group I, ^‡^p<0.05 versus group II, CKD: chronic kidney disease; S: serum; eNOS: endothelial nitric oxide synthase.

Serum biomarkers	Group I	Group II	Group III	(F)-value	p-value
S. adiponectin (µg/mL)	33.285±3.38	38.62±2.15*	46.36±2.72^†,‡^	42.36	0.032
S. leptin (ng/mL)	1.22±3.42	1.62±2.18*	1.94±3.29^†,‡^	32.42	0.041
S. cystatin C (ng/mL)	15.8±0.41	19.2±0.64*	21.3±0.87^†,‡^	57.55	0.028
S. eNOS (pg/mL)	757.5±0.56	707.5.5±0.65*	650±0.82^†,‡^	74.14	0.022

The results showed that serum adiponectin, leptin, and cystatin C levels were significantly higher in group II and group III patients compared with those in group I. In contrast, the serum levels of eNOS were significantly lower in group II and group III patients when compared with group I. These differences were found to be statistically significant (p<0.05).

Table 5 presents the correlation between eGFR and the studied serum biomarkers across the different CKD groups.

**Table 5:** This presents the correlation between serum adiponectin, cystatin C levels in patients with CKD Data are presented as mean ± standard deviation. Pearson’s correlation regression test was applied. S: serum; CKD: chronic kidney disease; (r)-value: Pearson coefficient; eGFR: estimated glomerular filtration rate.

Parameters	S. Adiponectin (r) -value	p-value	S. cystatin C (r) -value	p-value
Group I: eGFR (60-90 mL/min/1.73 m²)	0.36	0.045	0.44	0.042
Group II: eGFR (15-59 mL/min/1.73 m²)	0.52	0.032	0.62	0.038
Group III: eGFR (<15 mL/min/1.73 m²)	0.82	0.042	0.85	0.033

In group I, a significant positive correlation was observed between eGFR and serum adiponectin and cystatin C levels (p<0.05). In group II and group III, eGFR showed a significant positive correlation with serum adiponectin, cystatin C levels (p<0.05).

Table 6 presents the correlation between the eGFR and the studied serum biomarkers across the different CKD groups.

**Table 6:** Correlation between serum leptin and eNOS levels in patients with CKD Data are presented as mean ± standard deviation. Pearson’s correlation regression test was applied. (r) -value: Pearson coefficient; S: serum; eGFR: estimated glomerular filtration rate; CKD: chronic kidney disease; eNOS: endothelial nitric oxide synthase.

Parameters	S. leptin (r) -value	p-value	S. eNOS (r) - value	p-value
Group I: eGFR (60-90 mL/min/1.73 m²)	0.24	0.216	-0.22	0.164
Group II: eGFR (15-59 mL/min/1.73 m²)	0.43	0.028	-0.56	0.025
Group III: eGFR (<15 mL/min/1.73 m²)	0.76	0.025	-0.72	0.027

In group I, a weak positive correlation was also found between eGFR and leptin levels, although this relationship was not statistically significant. In group II and group III, eGFR showed a significant positive correlation with serum leptin, levels (p<0.05). Conversely, serum eNOS levels demonstrated a negative correlation with eGFR across the study groups. The correlation coefficients for group III, group II, and group I were r = −0.72, −0.56 and −0.22, respectively. This negative correlation was statistically significant in group III and group II, whereas the association in group I was not statistically significant.

## Discussion

The present study evaluated serum adipocytokine levels in patients with CKD and investigated their relationship with endothelial dysfunction. From Table 1, it was observed that patients in groups II and III had higher rates of smoking history, systolic and diastolic blood pressure, and BMI, compared with those in group I, and these differences were statistically significant. From Tables 2 and 3, it was evident that there was a clear inverse association between RBS levels and eGFR, indicating that poor glycemic control may contribute to worsening renal function. This observation was further supported by a concurrent increase in serum creatinine levels with rising RBS values, suggesting progressive renal impairment [15]. These findings of the present study are in accordance with those reported by Kumar et al., who described the biochemical mechanisms underlying hyperglycemia-induced vascular and renal damage [16].

In the present study, Table 4 showed that serum adiponectin levels increased significantly, with the highest concentrations observed in group III (stage V CKD). This increase may be attributed to the reduced renal clearance of adiponectin in patients with impaired kidney function [17]. The correlation analysis presented in Table 5 further demonstrated a strong relationship between adiponectin levels and declining renal function, with correlation coefficients of r =0.82, 0.52, and 0.36 in groups III, II, and I, respectively. These findings indicate that serum adiponectin levels tend to rise as renal function deteriorates. Similar observations have been reported in a previous study, which demonstrated an inverse relationship between adiponectin levels and eGFR, suggesting that elevated adiponectin concentrations may reflect worsening renal impairment [18]. Adiponectin is an adipocytokine predominantly secreted by white adipose tissue and is known to exert several protective biological effects. It improves insulin sensitivity through its antidiabetic action, reduces oxidative stress by inhibiting nicotinamide adenine dinucleotide phosphate (NADPH) oxidase activity, and suppresses inflammatory responses by downregulating pro-inflammatory cytokines [19]. In the present study, the analysis of Tables 3 and 4 demonstrated that serum creatinine and serum cystatin C levels increased significantly with the progressive decline in renal function, with the highest concentrations observed in group III (stage V CKD).

In the present study, as shown in Table 4, serum cystatin C levels were elevated in groups II and III when compared with group I suggesting a progressive increase in relation to renal function decline. As shown in Table 5, the correlation analysis revealed a strong association between serum cystatin C levels and declining eGFR, with correlation coefficients of r= 0.85, 0.62, and 0.44 in groups III, II, and I, respectively. The results suggest that serum cystatin C levels increase in parallel with worsening renal impairment. This observation is consistent with the findings of Benoit et al., who demonstrated that cystatin C is a sensitive marker for detecting subtle changes in eGFR [20].

The previous study by Shimizu et al. also reported that serum cystatin C serves as a highly sensitive biomarker for the early detection of alterations in glomerular filtration, particularly in patients with type 2 diabetes mellitus [21]. Table 5 indicates a significant association between increased cystatin C levels and CKD severity, accompanied by reduced eGFR. This is in agreement with findings by Sagar et al., who emphasized the value of cystatin C as an indicator of renal dysfunction [2,22]. Our findings demonstrate a stronger correlation between cystatin C concentrations and eGFR in patients with renal disease, consistent with previous studies by Qiu et al. [23]. Collectively, these findings emphasize the diagnostic value of cystatin C as a sensitive biomarker for detecting and monitoring CKD, particularly in individuals with type 2 diabetes mellitus [2].

In the present study, Table 4 demonstrated that serum leptin levels increased significantly with the progressive decline in renal function, with the highest concentrations observed in group III (stage V CKD). The correlation analysis showed that there was a positive association between serum leptin levels and declining eGFR, with correlation coefficients of r = 0.76, 0.43, and 0.24 in groups III, II, and I, respectively. These findings suggest that serum leptin concentrations increase in parallel with worsening renal impairment. The present study also indicated that CKD occurred more frequently among male patients with elevated serum leptin levels. Similar findings have been reported by Park et al. who observed that higher plasma leptin concentrations were positively associated with the incidence of CKD [24]. These study findings are in accordance with previous studies by Tondare et al. [7] and Ofori et al. [25]. Leptin has been shown to stimulate the sympathetic nervous system, which can contribute to sustained elevations in blood pressure and subsequent renal injury. In addition, reduced renal clearance alone may not fully explain the increased leptin levels observed in CKD. Another study by Korczynska et al. [26] reported increased expression of the leptin (LEP) gene in the subcutaneous adipose tissue of patients with CKD compared with healthy individuals. In addition, leptin mRNA levels in adipose tissue were found to correlate positively with circulating leptin concentrations. These findings suggest that enhanced leptin production by adipocytes may contribute to the hyperleptinemia frequently observed in CKD patients. Accumulating evidence from both in vivo and in vitro studies further supports a mechanistic relationship between elevated leptin levels and endothelial dysfunction [27].

In the present study, endothelial dysfunction was assessed by evaluating plasma nitric oxide (NO) levels through the measurement of eNOS. Table 6 revealed a strong negative correlation between eGFR and serum eNOS levels across the study groups, with correlation coefficients of r = −0.72, −0.56, and −0.22 in groups III, II, and I, respectively. These findings indicate that eNOS levels decline progressively as renal function deteriorates. The present findings are in agreement with prior studies that have demonstrated diminished nitric oxide bioavailability in individuals with CKD [28]. The decrease in NO levels may be attributed to several mechanisms, including reduced renal synthesis of L-arginine, increased arginase activity, and impaired function of renal and endothelial nitric oxide synthase (nNOS-α) [29]. These alterations may contribute to endothelial dysfunction and the increased cardiovascular risk commonly observed in patients with CKD.

The strength of the present study lies in its contribution to the understanding of the interrelationship between adipocytokines and eNOS and their potential involvement in the pathophysiology of CKD. The limitations of the present study are that it is a relatively small sample size and a cross-sectional, single-center design study.

## Conclusions

The findings of the present study indicate that CKD is associated with significant alterations in adipokine concentrations as a consequence of progressive deterioration in renal function. These changes become more pronounced in the advanced stages of CKD. A significant relationship was observed between adipokine levels and endothelial function, suggesting that the interaction among these factors may contribute to increased cardiovascular risk even during the pre-dialysis stages of CKD. Although the serum cystatin C assay is relatively more expensive than conventional serum creatinine testing, it offers greater diagnostic accuracy, particularly for the early detection of renal impairment. Therefore, routine assessment of serum cystatin C may be beneficial for the early identification of CKD in high-risk populations, potentially helping to prevent complications and improve patient outcomes. 
